# Crack Detecting Method Based on Grid-Type Sensing Networks Using Electrical Signals

**DOI:** 10.3390/s23136093

**Published:** 2023-07-02

**Authors:** Ju-Hun Ahn, Yong-Chan Lee, Se-Min Jeong, Han-Na Kim, Chang-Yull Lee

**Affiliations:** 1 Department of Aerospace Engineering, Inha University, Incheon 22212, Republic of Korea; 2 Department of Aerospace Engineering, Chosun University, Gwangju 61452, Republic of Korea; 3 Department of Naval Architecture and Ocean Engineering, Chosun University, Gwangju 61452, Republic of Korea

**Keywords:** electrohydrodynamics, crack detecting, micro-crack sensor, conductive ink

## Abstract

Cracks have a primary effect on the failure of a structure. Therefore, the development of crack sensors with high accuracy and resolution and cracks detection method are important. In this study, the crack sensors were fabricated, and the crack locations were detected with the electrical signal of the crack sensor. First, a metal grid-type micro-crack sensor based on silver was fabricated. The sensor is made with electrohydrodynamics (EHD) inkjet printing technology, which is well known as the next generation of printed electronics technology. Optimal printing conditions were established through experiments, and a grid sensor was obtained. After that, single cracks and multiple cracks were simulated on the sensor, and electrical signals generated from the sensor were measured. The measured electrical signal tracked the location of the cracks in three steps: simple cross-calculation, interpolation, and modified P-SPICE. It was confirmed that cracks could be effectively found and displayed using the method presented in this paper.

## 1. Introduction

Cracks are caused by continuous loads and extreme temperature circumstances, and depending on the cracks’ location, inner and outer crack can occur [1]. Cracks initiated from the outside are visible to the naked eye. However, in the case of cracks starting from the inside, it is impossible to check with the naked eye, so techniques such as non-destructive testing must be used. For structures where the crack occurs, the strain energy is greater than that of the non-crack-producing structures, which in turn makes the critical load intolerable [2]. Cracks also cause serious structural problems, including corrosion [3,4]. In the case of bridges, the load on those parts can be concentrated and broken due to the cracks, and the cracks in the pipeline that moves the gas can cause internal corrosion and deformation, resulting in a serious disaster that explodes [5]. In addition to bridges and gas pipes, vehicles operating long distances, such as aircraft and ships, are also sensitive to cracking. For aircrafts, the lowest ambient air temperature in flight is −50 °C, which is derived from very extreme temperature environments, making them vulnerable to cracking. Cracks on these aircraft can also cause resistance and, in severe cases, damage to the fuselage and wings can lead to falls. Cracks in these various structures are important defects that leads to serious damage and methods for their detection are being developed in various ways, starting with visual inspections [6]. However, detecting cracks in all parts of the structure requires considerable time and resources [7].

Microcracks are a factor in the accumulation of fatigue damage in structures and are the starting point for the development of large cracks, as stress is concentrated at the point where microcracks occur. Therefore, they are being studied along with large-scale cracks [8,9,10,11]. Because the interactions between microcracks and macro-cracks cause defects such as voids, cracks, and inhomogeneities, research on microcrack detection is needed [10]. Sensor fabrication and integration mostly depend on the semiconductor manufacturing methods and equipment, which makes it difficult to realize a sensor array on a large-area substrate and incurs limitations in fabricating a sensor with a flexible and uneven surface [11]. Inkjet printing technology has the advantage of being able to be arranged in a desired manner [12].

Structural health monitoring (SHM) is a technology studied to solve these problems and periodically check information such as structural damage caused by loads and cracks, as well as system health checks [13]. In addition, rapid monitoring is possible even in situations such as earthquakes or unexpected explosions [14,15]. SHM applies simple verification methods such as direct monitoring, tapping echo, acoustic emission testing, and thermal imaging, and various methods are being studied for clearer monitoring [16], examples of which include fiber bragg grating (FBG) sensor technology, electroluminescent strain sensor technology, and image-based crack detection technology. The FBG method is an SHM method that uses ultrasonic waves to determine the degree of damage [15]. Many studies have been conducted using and applying SHM. Zhao et al. studied SHM using FBG and found that when FBG was operated, numerous temperature changes and low frequency noise occurred in the measurement of the structure’s ultrasonic wave induction (UGW). Temperature changes and low frequency noise pose challenges in understanding real-world structural changes, and to overcome these problems, the author studies adaptive wavelength control systems using acoustic emission and ultrasound [17]. Electroluminescent strain sensor technology visualizes and verifies the strain and stress of a structure, and Xu and Jo proposed a precisely controllable Wheatstone bridge and monochrome electroluminescence (EL) technology for the dynamic detection of a structure [18]. In addition, strain sensors have the characteristics of being manufactured as flexible sensors, and crack-based strain sensors have been studied [19,20]. Finally, for image processing technology, Lins at al. studied image-based crack detection technology that detects cracks through image processing [21]. SHM is being applied to many technologies [22].

Electrohydrodynamics (EHD) inkjet printing technology is a technology that enables micro-level ejection using electrical power. In this printing technology, the electric field formed between the nozzle and the substrate breaks the surface tension of the ink, and it is possible to eject a thickness smaller than the inner diameter of the nozzle [23,24]. In addition, since the printing resolution is more than twice as high as the existing inkjet printing technology, it is a suitable technology for manufacturing micro-crack sensors with high accuracy. Therefore, EHD inkjet printing technology is the latest printing technology being studied in various fields [25,26,27].

In this study, a metal grid crack sensor was fabricated using EHD inkjet printing. Prior to the full-scale experiment, to find the optimum value of the experimental parameter of the conductive ink used in sensor manufacturing, an optimization test was conducted through EHD inkjet printing, and metal-grid printing was applied according to the test results. A metal-grid crack sensor was manufactured using three samples according to the location, direction, and number of cracks, and has a 10 × 10 grid scale. To estimate the location of the crack, the rate of change in resistance was measured and applied to algorithms using simple cross-calculations and the interpolation method. Furthermore, to use the above methods, the resistance change rates were crossed and overlapped, however, by filtering the measurement using Modified-P-SPICE, the location of the cracks was more intuitive.

## 2. Electrohydrodynamics Technology

### 2.1. Principle of EHD Inkjet Printings

**Figure 1:**
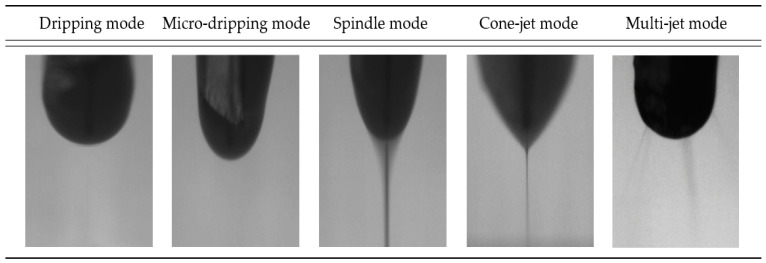
The ejection mode of EHD inkjet printing system.

EHD inkjet printing is a method of printing conductive ink through an electric field created between a nozzle and a substrate. When an electric field is not formed between the nozzle and substrate, ink is formed in a hemispherical shape at the tip of the nozzle. At this moment, by applying a voltage to the electrostatic force-based nozzle to form an electric field with the substrate, ions of the same polarity are focused on the meniscus surface, and as the applied voltage increases, the surface tension of the ink is broken; thus, the conductive ink is printed. Hence, during printing, lines thinner than the inner diameter of the nozzle are printed, and the printing mode is determined depending on the voltage level. In EHD inkjet printing, the ejection mode is determined through experimental parameters such as the ink characteristics, voltage, flow rate, and ejection height. In EHD inkjet printing, the ejection mode is determined through experimental parameters such as the ink characteristics, voltage, flow rate, and ejection height. Figure 1 represents the EHD inkjet printing ejection modes. The dripping mode is generated by gravity rather than the effect of an electric field. As the voltage increases, the meniscus at the tip of the nozzle decreases. The micro-dripping mode is similar to the dripping mode, but the droplets are smaller than the dripping mode, and it mainly occurs at higher voltage. The spindle mode is printed into a column shape. At the same voltage, when the flow rate is over-applied, the ink is printed into smaller droplets, and satellite droplets are printed around the array, making it difficult to print stably. In the Cone-jet mode, also known as Taylor-cone mode, because the tip of the meniscus is finely discharged, the printing meniscus can be shown most stably in a conical shape; therefore, Cone-jet mode is generally used for EHD inkjet printing technology. Finally, in multi-jet mode, when the applied voltage exceeds the standard, the conductive ink is diffused from the nozzle tip and printed.

### 2.2. EHD Inkjet Printing System

**Figure 2:**
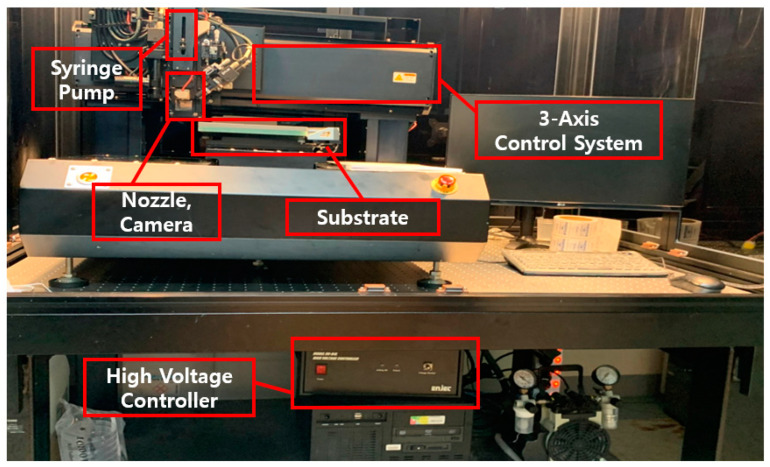
Equipment composition and designation of EHD printing systems.

To accurately and stably print using EHD inkjet printing, a process variable control module is required. Figure 2 shows the EHD inkjet printing system. The flow controller applies constant pressure to the conductive ink in the syringe such that the amount specified by the user can be uniformly discharged. The high-voltage amplifier creates an electric field between the substrate and the nozzle; therefore, the conductive ink is discharged into a size smaller than the inner diameter of the nozzle. The three-axis transfer device prints a user-designated pattern by controlling the *x*, *y*, and *z* axis, and enables a stable discharge by adjusting the printing height. In addition, high-speed cameras and LED lights are applied that allow the user to check the discharge shape in real time through the display. In addition, vacuum pumps are used to fix the film and glass plate on the substrate. These modules provide complete process control through software and can be controlled by the user through the display.

## 3. Fabrication Process of Metal-Grid Samples

### 3.1. Ink Production Process

**Figure 3:**
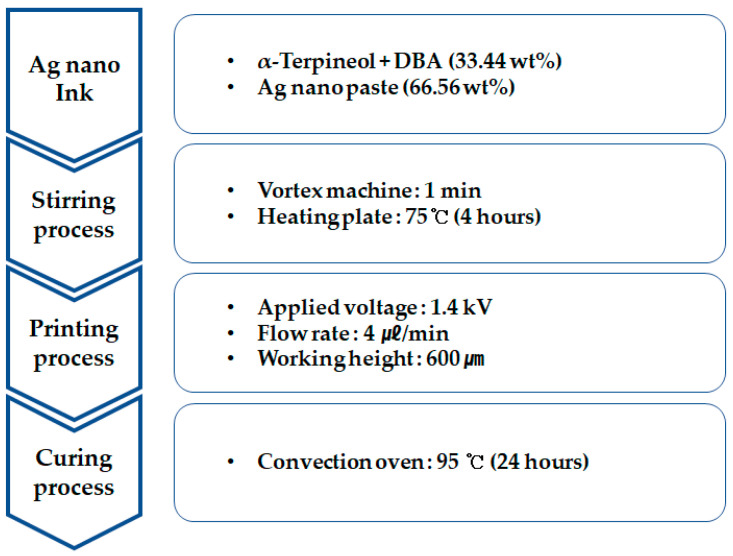
Sample production process through EHD inkjet printing experiment.

Figure 3 shows a schematic diagram of an experimental method used for preparing a metal grid sample. Silver nano ink is a compound that contains silver nano paste and solvent. The solvent was produced using α-terpineol, which is a surfactant agent, and diethylene glycol monobutyl ether acetate (DBA), which is a nonpolar solvent. Then, 66.56 wt% silver nano paste and 33.44 wt% of the prepared solvent were mixed. Silver nano-ink was dispersed for 4 h using a stirrer to produce metal-grid samples.

### 3.2. Jetting Optiomization of Conductive Ink Using EHD Inkjet Printing

To print conductive ink using an EHD inkjet printing system, it is essential to confirm the optimal conditions for the experimental parameters such as the printing speed, flow rate, and applied voltage. Thus, the optimum speed for the sample production was derived by determining the thickness of the circuit at each printing speed.

**Figure 4:**
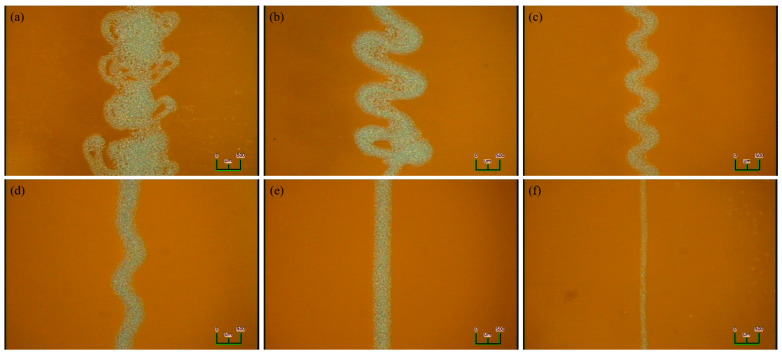
Jetting status and discharge thickness based on the EHD printing speed: (**a**) 10 mm/s, (**b**) 120 mm/s, (**c**) 130 mm/s, (**d**) 150 mm/s, (**e**) 160 mm/s, (**f**) 500 mm/s.

Figure 4 shows an image of the jetting status and the thickness at each printing speed. Because this is an experiment to measure the thickness according to the printing speeds, the values of the applied voltage, flow rate, and jetting height were fixed as follows. The applied voltage was 1.4 kV, the flow rate was 4 μL/min, and the jetting height was 600 μm. The printing speed was 10–500 mm/s, and the jetting conditions and thickness of the printed lines were checked at each speed. From 10 to 110 mm/s, the printing speed was quite slow, and irregular whipping patterns were confirmed. The thickness of the printed line where whipping occurred was found to have various line widths ranging from 150 to 950 μm at a low speed. In addition, the printed ink slightly overlapped at the center of the line because of whipping. At a printing speed of 120 mm/s, the pattern was confirmed to be slightly more periodic than the whipping phenomenon that occurred at a lower speed. The thickness range was 250–650 μm, and it was confirmed that the thickness also gradually decreased as the printing speed increased. At a printing speed of 130–140 mm/s, the whipping shape was periodically verified, and the thickness of the printed line was approximately 400 μm. At a printing speed of 150 mm/s, it tended to be similar to the previous speed with a thickness of 400 μm, but it seemed like the spread and frequency of whipping decreased. At 160 mm/s, whipping did not occur, and stable jetting was conducted, and it seemed as if it was printed in a straight line of 400 μm. At 170–500 mm/s, the thickness gradually decreased to 400 μm or less, and at 500 mm/s, it seemed as if it was printed up to 100 μm. Therefore, to prepare the samples, the printing speed should be set to 160 mm/s or more. However, considering that the tip of the probe pin for the measurement is 100 μm, the sample was printed at 280 mm/s for a stable thickness of 300 μm.

### 3.3. Crack Sample Case

**Figure 5:**
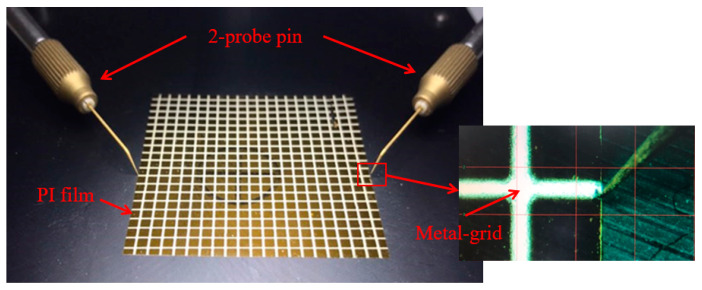
Fabricated crack sensor and the measurement method.

**Figure 6:**
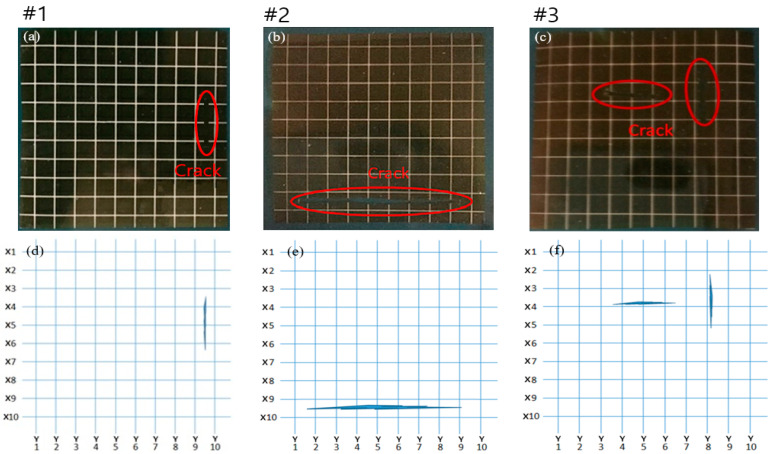
10 × 10 grid type crack sample cases: (**a**) sample #1, (**b**) sample #2, (**c**) sample #3, (**d**) parenting diagram of sample #1, (**e**) parenting diagram of sample #2, and (**f**) parenting diagram of sample #3.

Silver nano-ink was patterned in a grid type on polyimide (PI) film, which has excellent mechanical properties and is mainly used for flexible sensors. The patterned PI film was cured at 95 °C for 24 h in a convection oven. Figure 5 shows the crack sensor fabricated by the method described above and the measurement method. For measurement, a probe pin was used to measure fine-sized lines. Resistance was measured by contacting both sides of the metal grid with this probe pin. Three cases of crack samples were prepared. Figure 6a,c show photographs of the samples, and Figure 6d,f show a schematic diagram of each case. The first crack is made in the middle of the right side of the PI film, as shown in Figure 6a,d. The second crack is in the middle of the lower part of the PI film, as shown in Figure 6b,e. The third case is multiple cracks, and the cracks are made in the middle of the PI film. The directions of the cracks are perpendicular to each other. The third case is shown in Figure 6c,f.

## 4. Results and Discussion

### 4.1. Resistance Chage by Crack Occurence

**Figure 7:**
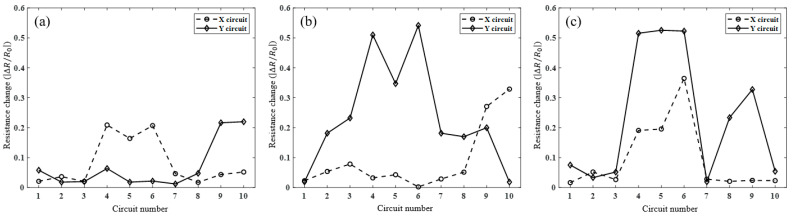
Ratio of changed resistance on the crack location of each sample: (**a**) sample #1, (**b**) sample #2, and (**c**) sample #3.

The resistance of each sample was measured by contacting the probe pin of the probe station at the end of each circuit, and the rate of resistance change was calculated by comparing the resistance of the non-cracked and cracked samples. Figure 7a shows the rate of resistance change for each circuit of the right crack sample composed of a 10 × 10 grid. In the X-direction circuit, the rates of resistance change were relatively large at X4, X5, and X6, and the measurements were 0.209, 0.164, and 0.207, respectively. In the Y-direction circuit, the resistance change rates at Y9 and Y10 were 0.217 and 0.220, respectively, and the resistance change rate was measured to be relatively large. At Y4 and Y8, it appears that the resistance change rate increased; however, in the non-cracked circuit, 0.064 and 0.048 are not relatively large, and thus, the noise was considered to be generated during the measurement. Figure 7b shows the rate of resistance change for each circuit of the crack sample at the bottom. In the X-direction circuit, the rates of resistance change at X9 and X10 were 0.282 and 0.331, respectively. In the Y-direction circuit, the rates of resistance change at Y2–Y9 were 0.191, 0.241, 0.506, 0.351, 0.551, 0.182, 0.170, and 0.204, respectively, and the rate of resistance change was measured as relatively large. Figure 7c shows the rate of change of resistance by the circuit for multiple crack samples. In the X-direction circuit, the rates of change in resistance at X4, X5, and X6 were measured as relatively large, and the measurement was 1.982, 2.044, and 3.574, respectively. In the Y-direction circuit, the rates of resistance change in Y4–Y6 and Y8 and Y9 were measured to be relatively large, and the measured values were 0.515, 0.525, 0.523, 0.234, and 0.328, respectively.

### 4.2. Crack Estimation Using Simple Cross-Calculation

**Figure 8:**
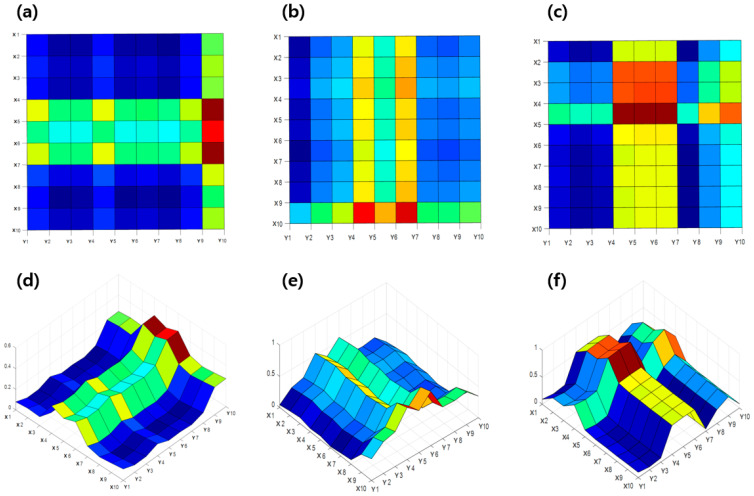
Location estimation results based on simple cross-calculation: (**a**) 2D version of sample #1, (**b**) 2D version of sample #2, (**c**) 2D version of sample #3, (**d**) 3D version of sample #1, (**e**) 3D version of sample #2, and (**f**) 3D version of sample #3.

Figure 8 shows the crack estimation through a simple cross-calculation of samples consisting of a 10 × 10 grid. Figure 8a,c are represented through 2D coordinates, and Figure 8d–f are expressed in 3D coordinates. Based on the previous results, the measurements of the X- and Y-directions were intersected and then overlapped to be expressed in 3D coordinates. The red spot of the coordinates was the largest among the values obtained by overlapping after the rate of resistance change crosses in the corresponding cracked circuit. Green/yellow spots are values of data with a large rate of resistance change owing to cracks in the X or Y circuit and data from the non-cracked circuit. The blue spots are parts of the values consisting only in a non-cracked circuit. For metal grid sample #1 in Figure 8a,d, the location of the crack was estimated to be in the vertical direction at the end of the right side. For sample #2, shown in Figure 8b,e, the crack was estimated to be in the horizontal direction at the bottom of the grid. However, for sample #3 in Figure 8c,f, which is the result of a multi-crack sample, it was difficult to accurately estimate the location of the crack through a simple cross-calculation of the measurements. As a result, although it is possible to estimate the location of a single crack through a simple intersection calculation, a slight error occurs because the range of the corresponding spots is rather wide. In the case of multiple cracks, it is difficult to accurately estimate their location.

### 4.3. Crack Estimation Using Interpolation

**Figure 9:**
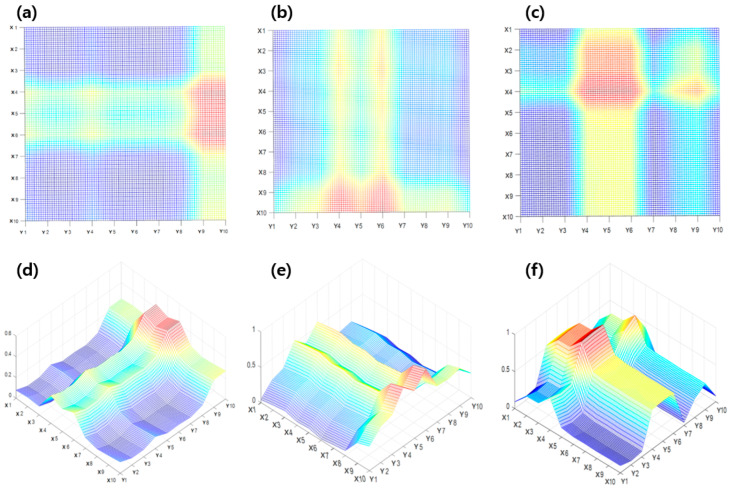
Location estimation results based on interpolation: (**a**) 2D version of sample #1, (**b**) 2D version of sample #2, (**c**) 2D version of sample #3, (**d**) 3D version of sample #1, (**e**) 3D version of sample #2, (**f**) 3D version of sample #3.

As the results in Figure 8 show, interpolation was considered as a method to supplement the defects of crack estimation from the simple cross-calculation. Figure 8 shows the crack estimation using the interpolation method of a metal-grid sample with cracks under three conditions. Similar to the simple crossover calculation, the red spot represents the rate of resistance change crossing/overlapping in the cracked circuit, and the green/yellow spot represents the part where the data on a large rate of resistance change from cracks and the data on the non-cracked circuit are combined. In addition, the blue spot represents a part made up of a non-cracked circuit. Figure 9a,c show 2D data obtained through the interpolation method, and Figure 9d–f show the 3D coordinates through the crossing/overlapping of the rate of resistance change for which the interpolation method is applied. Figure 9a,d show the results of the interpolation method indicated in Figure 8a,d. Figure 9a,d show estimations of the crack in the vertical direction at the end of the right side, as in the previous result, and the approximate crack size can be confirmed. Figure 9b,e show the results of the interpolation method in Figure 8b,e. In addition, as in the previous results, the horizontal crack at the bottom of the grid was estimated, and the size of the crack was approximately confirmed. Figure 9c,f show the results of the interpolation method of Figure 8c,f. It was possible to estimate multiple cracks that could not be confirmed in the previous simple crossover estimation results, and two cracks were successfully estimated. Compared with the simple cross-calculation, the interpolation method was more accurate for estimating the crack location because the grid scale was elaborate. Therefore, it was possible to estimate multiple cracks and the approximate crack size, which was difficult to estimate in a simple cross-calculation.

### 4.4. Crack Estimation Using Modified P-SPICE

The coordinates obtained in Figure 8 and Figure 9 are also expressed by overlapping the values from the non-crack circuit. For each of the previously measured samples, the tendency of the resistivity change rate due to the crack overlapped was obtained, but for this sample, the data intersecting the resistance change rate by circuit was filtered to enable intuitive position estimation, which highlighted the range of crack locations. In addition, by filtering the data from the location where the resistance change rate from cracks and the rate of resistance change in a circuit without cracks overlap, we tried to obtain the intuitiveness of the crack location coordinates. The trend of the filtered rate of resistance change is displayed in three dimensions, and at the same time, the estimated crack location can be checked.

**Figure 10:**
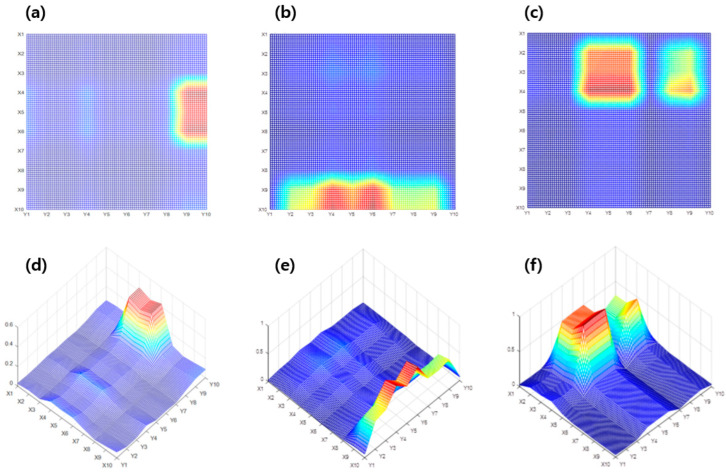
Location estimation results for each corrected sample using Modified-P-SPICE: (**a**) 2D version of sample #1, (**b**) 2D version of sample #2, (**c**) 2D version of sample #3, (**d**) 3D version of sample #1, (**e**) 3D version of sample #2, and (**f**) 3D version of sample #3.

Figure 10 shows the coordinates of the rate of resistance change for each sample applied with a correction factor through Modified-P-SPICE. As shown in Figure 10a,d, the 2D and 3D coordinates are obtained by filtering the position estimate coordinates from sample #1 through the correction coefficients. As confirmed in Figure 7 and Figure 8, the overlapping rates of resistance change in the existing non-crack circuit are included in such a way that a certain part is distinguishable based on the color of the contour; however, a correction is required for the intuitive part. The crack estimation range was emphasized by filtering a certain portion of the rate of resistance change in the non-crack through the correction factor. It can be estimated that cracks are generated between X4, X5, X6, Y9, and Y10 in Figure 10a,d. The resistance is measured through the ends of each circuit. Because the grid is a bit wider than the actual size of the crack, the approximate location and range can be known. Figure 10b,e show the 2D and 3D coordinates obtained by filtering the location estimate coordinates for sample #2 through a correction factor. Figure 10c,f are also 2D and 3D coordinates filtered by the correction factor from the positional coordinates for sample #3.

## 5. Conclusions

In this study, using EHD inkjet printing technology, the reliability of the location estimation algorithm was examined through various crack cases for the detection of microcracks. After measuring the resistance change, the crack location was estimated using the tendency of the rate of resistance change of the part where the crack occurred, and different 2D and 3D crack estimation coordinates were confirmed through three crack cases. In the case of a single crack and multiple cracks in the lateral direction, although they were in a wide range, the location information for the corresponding cracks was obtained. However, in the case of multiple cracks in the longitudinal direction, the noise in the rate of resistance change was severe, and thus, the location estimation range was ambiguous. If the grid scale increases to 20 × 20 or 50 × 50 instead of 10 × 10, the accuracy of the location and the direction of the cracks is considered to improve via the precise grid configuration. Also, data increases as the scale increases. At this time, the modified P-SPICE method is expected to be able to quickly and accurately detect the position of cracks.

## Data Availability

The data are available upon request from the corresponding author.

## References

[1] Giurgiutiu V., Xu B., Liu W. (2010). Development and testing of high-temperature piezoelectric wafer active sensors for extreme environments. Struct. Health Monit..

[2] Brayshaw W.J., Cooper A.J., Sherry A.H. (2019). Assessment of the micro-mechanical fracture processes within dissimilar metal welds. Eng. Fail. Anal..

[3] Li W., Chen G., Yin X., Jiang W., Zhao J., Ge J. (2019). Inspection of both inner and outer cracks in aluminum tubes using double frequency circumferential current field testing method. Mech. Syst. Signal Process..

[4] DuQuesnay D.L., Underhill P.R., Britt H.J. (2003). Fatigue crack growth from corrosion damage in 7075-T6511 aluminium alloy under aircraft loading. Int. J. Fatigue.

[5] Javidi M., Horeh S.B. (2014). Investigating the mechanism of stress corrosion cracking in near-neutral and high pH environments for API 5L X52 steel. Corros. Sci..

[6] Bhuiyan M.Y., Bao J., Poddar B., Giurgiutiu V. (2018). Toward identifying crack-length-related resonances in acoustic emission waveforms for structural health monitoring applications. Struct. Health. Monit..

[7] Sahay P., Kaya M., Wang C. (2013). Fiber Loop Ringdown Sensor for Potential Real-Time Monitoring of Cracks in Concrete Structures: An Exploratory Study. Sensors.

[8] Li H., Jiang W., Deng J., Yu R., Pan Q. (2022). A Sensitive Frequency Range Method Based on Laser Ultrasounds for Micro-Crack Depth Determination. Sensors.

[9] Alam S.Y., Loukili A. (2020). Effect of micro-macro crack interaction on softening behaviour of concrete fracture. Int. J. Solids Struct..

[10] Soh A.K., Yang C.H. (2004). Numerical modeling of interactions between a macro-crack and a cluster of micro-defects. Eng. Fract. Mech..

[11] Shin J., Lee W.H., Nothnagle C., Wijesundara M.B. (2014). EHD as sensor fabrication technology for robotic skins. In Next-Generation Robots and Systems. Int. J. Opt. Photonics.

[12] Azar G.T.P., Danilova S., Krishnan L., Fedutik Y., Cobley A.J. (2022). Selective Electroless Copper Plating of Ink-Jet Printed Textiles Using a Copper-Silver Nanoparticle Catalyst. Polymers.

[13] Mora B., Basurk J., Sabahi I., Leturiondo U., Albizuri J. (2023). Strain Virtual Sensing for Structural Health Monitoring under Variable Loads. Sensors.

[14] Farrar C.R., Worden K., Arnaud D., Keith W. (2010). An Introduction to Structural Health Monitoring. New Trends in Vibration Based Structural Health Monitoring.

[15] Amano M., Okabe Y., Takeda N., Ozaki T. (2007). Structural health monitoring of an advanced grid structure with embedded fiber Bragg grating sensors. Struct. Health. Monit..

[16] Wu C., Sun K., Xu Y., Zhang S., Huang X., Zeng S. (2019). Concrete crack detection method based on optical fiber sensing network and microbending principle. Saf. Sci..

[17] Zhao Y., Zhu Y., Yuan M., Wang J., Zhu S. (2016). A laser-based fiber Bragg grating ultrasonic sensing system for structural health monitoring. IEEE Photon. Technol. Lett..

[18] Park J., Kim D.S., Yoon Y., Shanmugasundaram A., Lee D.W. (2023). Crack Crack-Based Sensor by Using the UV Curable Polyurethane-Acrylate Coated Film with V-Groove Arrays. Micromachines.

[19] Kim Y., Park C., Kim J., Kim H., Park C., Lee B., Jeong Y., Cho S.J. (2020). Effects of bending strain and crack direction on crack-based strain sensors. Smart. Matrer. Struct..

[20] Xu J., Jo H. (2015). Development of high-sensitivity and low-cost electroluminescent strain sensor for structural health monitoring. IEEE Sens. J..

[21] Lins R.G., Givigi S.N. (2016). Automatic crack detection and measurement based on image analysis. IEEE Trans. Instrum. Meas..

[22] Jian-guo N., Hui-lan R., Min-jie F. (2014). Research on the process of micro-crack damage evolution and coalescence in brittle materials. Eng. Fail. Anal..

[23] Ahn J.H., Choi J.H., Lee C.Y. (2020). Electrical evaluations of anisotropic conductive film manufactured by electrohydrodynamic ink jet printing technology. Org. Electron..

[24] Hassan R.U., Khalil S.M., Khan S.A., Ali S., Moon J., Cho D.H., Byun D. (2022). High-Resolution, Transparent, and Flexible Printing of Polydimethylsiloxane via Electrohydrodynamic Jet Printing for Conductive Electronic Device Applications. Polymers..

[25] Raje P.V., Murmu N.C. (2014). A review on electrohydrodynamic-inkjet printing technology. Int. J. Emerg. Technol. Adv. Eng..

[26] Lee K.I., Lim B., Lee H., Kim S.H., Lee C.S., Cho J.W., Chung S., Hong Y. Multi nozzle electrohydrodynamic inkjet printing head by batch fabrication. Proceedings of the 2013 IEEE 26th International Conference on Micro Electro Mechanical Systems (MEMS).

[27] Lyu H., Zhang X., Liu F., Huang Y., Zhang Z., Jiang S., Qin H. (2019). Fabrication of micro-scale radiation shielding structures using tungsten nanoink through electrohydrodynamic inkjet printing. J. Micromech. Microeng..

